# Fiat Justitia, Ruat Cœlum

**Published:** 1903-01

**Authors:** 


### FIAT JUSTITIA, RUAT CŒLUM.

THE child of a man named C. C. Justice, residing at Dublin, fell sick. Justice being bit by the Christian Science heresy refused to call in a physician, or to administer the necessary remedies, so that the child was translated to that sphere where wrestling in prayer with the "mortal mind" becomes superfluous. The well-disposed and outraged neighbors piled Pelion on Ossa by calling for justice on Justice, and lodged complaint against him, alleging that the child had died from criminal neglect. The local judge imposed a fine on Justice of $300, and it appeared that justice was satisfied. She was, but he wasn't. The case was appealed to the Supreme Court, and the learned judge, Little, J., reversed the decision of the lower court in giving the opinion that failure to administer medicine to a minor was not within the legal interpretation of withdrawing sustenance. It might be stated in other words that medicine is not a food—a contention that may well be passed without argument. The Christian Scientists regard this decision of "Little, J.," as a grand triumph of truth—and Justice, and a telling defeat for the sons of Belial and the seed of the Amalekite. But it would be interesting to know wherein lies either the triumph or the defeat. If it is a triumph to Christian Science that legally a man may allow his child to die when it could have been saved, they are certainly free to enjoy the fruits of it. It is not quite apparent how the preventable death of a child vindicates or establishes the truth of Christian Science. To one "mortal mind" it merely adds an element of intolerable cruelty and brutality to a belief which is otherwise contemptibly amusing. Do they imagine that this decision will close the drug stores and cause the physician to cease from his labors in the relief of suffering and the prolongation of life?

Christian Science shows ever clearer signs of disintegration. The Eddyites are trimming and hedging and eagerly grasping at any support that will keep them a little longer from submergence and disappearance. When they do sink beneath the waters of oblivion nothing will be left but bubbles that, bursting, give forth a mephitic odor.

